# Acknowledgment to Reviewers of *Medicina* in 2020

**DOI:** 10.3390/medicina57020086

**Published:** 2021-01-21

**Authors:** Medicina Editorial Office

**Affiliations:** MDPI AG, St. Alban-Anlage 66, 4052 Basel, Switzerland

Peer review is the driving force of journal development, and reviewers are gatekeepers who ensure that *Medicina* maintains its standards for the high quality of its published papers. Thanks to the cooperation of our reviewers, in 2020, the median time to first decision was 22 days and the median time to publication was 40 days. The editors would like to express their sincere gratitude to the following reviewers for their precious time and dedication, regardless of whether the papers were finally published:
A. H. Järvinen, TeroLevanon, KerenAasly, JanLevesque, EricAbbate, VincenzoLevi Sandri, Giovanni BattistaAbbate-Daga, GiovanniLevis, MarioAbel, ThomasLi, PingAberer, FelixLi, ShuoAchimas, PatriciuLi, WeiAchiron, AsafLi, WenboAdamczewska, KatarzynaLibera, Dacia DallaAddison, CliftonLicari, LeoAdekoya, Timothy O.Licini, CaterinaAdonakis, GeorgeLieb, John G.Adriani, AlessandroLieneck, CristianAdur, MalavikaLim, Sang MooAfrashtehfar, Kelvin IanLin, Chao-NanAfshar, YaldaLindemann, StephanAggarwal, ManishLinder, MarieAgresta, FerdinandoLindroth, HeidiAguglia, AndreaLinek, PawełAguiar, PatrícioLipphaus, AndreasAhlander, Britt-MarieLippolis, AntonioAhmad, AsrarLisowska, BarbaraAhmad, TanveerLitofsky, Norman ScottAi, YongLiu, ChenAik, JoelLiu, David T.Aja, AravamudhanLiu, LukaiAkima, HiroshiLiu, MengyangAksan, AysegulLiu, XiuyunAlaimo, AlessandroLiu, Yen BinAlam, Mohammad ParvezLiu, YingpengAlam, SaminaLo Gullo, AlbertoAlam, ShafiulLo, ChristopherAlbert, StewartLobysheva, IrinaAlbizua, IgorLocatelli, FrancescoAlderman, JanLofrumento, Dario D.Alessio, GambinoLoiudice, PasqualeAlexa-Stratulat, TeodoraLombardo, MauroAl-Ghabkari, AbdulhameedLondero, Ambrogio P.Ali, SarvathLong, TaoAlkilany, ReemLongo, MicheleAllegaert, KarelLopez Navas, Ana I.Allen, JyaiLópez, DanielAlloza, IraideLópez-Gil, José FranciscoAlmeida-Santos, Ana TeresaLópez-Sáez, Juan BoscoAloisi, Anna MariaLordkipanidzé, MarieAlwahsh, SalamahLorenzo, JuliaAmano, ToshiyasuLorenzo, MicheleAmenta, FrancescoLosurdo, GiuseppeAmin, KawaLouis, CorentinAmmerman, SethLuangmonkong, TheerutAmornyotin, SomchaiLubetzky, Anat V.Amzar, DanielaLucas, JosephAnastasiadou, EleniLucchese, AlessandraAndabak Rogulj, AnaLuchini, ClaudioAnderson, Greg M.Luchtmann, MichaelAndo, HitoshiLudy, Mary-JonAndrews, Shea J.Lukacs, ZoltanAnghel, LarisaLundh Hagelin, CarinaAnichini, RobertoLupo, MichelaAnsa, BenjaminLusczek, Elizabeth R.Antal, OanaŁuszczki, EdytaAntoniou, EvangeliaLuzak, BoguslawaAntoniou, MichaelLyhne, Mads DamAntonoglou, Georgios N.Machalinski, AnnaAntón-Solanas, IsabelMachuca-Portillo, GuillermoAnzivino, RobertaMackrill, JohnApostolakis, SotiriosMadka, VenkateshwarApostolopoulos, NikosMadzvamuse, AnotidaArai, RyuzoMagafas, LykourgosArakaki, TatsuyaMagan-Fernandez, AntonioAranha, Ágata MarquesMagnano San Lio, RobertaArif, TasleemMagnus, PerArmengol, José ÁngelMahieu, Ludo M.Armstead, William M.Majewska, AnnaArmstrong, NeilMaji, DebnathArnason, GardarMalaguarnera, MicheleArora, HarneetMalham, MikkelArora, KomalMalli, FoteiniArráez-Aybar, Luis-AlfonsoMallineni, Sreekanth KumarArshad, NajlaMaloberti, AlessandroAshrafian Bonab, MaziarMalvè, MauroAslanidis, TheodorosMamiya, Ping C.Atkinson, ArielMamouni, KenzaAttia, SamehManach, Yannick LeAtzori, LauraMandal, AbhirupAuerbach, MichaelMandelbaum, DavidAuffarth, Gerd U.Mandic, RobertAurora, RajeevMandurino-Mirizzi, AlessandroAvadhani, VaidehiMangaonkar, AbhishekAvilés, Francesc XavierMannino, DavidAvino, AdelaidaManolopoulos, AnnaAwada, HassanMansilla, Juan RodríguezAyoade, FolusakinMånsson, AlfBaba, YuhMansueto, GiancarloBacci, ChristianMantadakis, ElpisBaccouche, AsmaMantica, GuglielmoBack, MichaelManyalich, MartíBado, IgorMarchese, MariaBaggieri, MelissaMarcos Pardo, Pablo JorgeBahji, AneesMarginǎ, DenisaBakht, MartinMaria Tsekoura, Maria TsekouraBakri, ElsheikhMarkiet, KarolinaBalaban, Daniel VasileMarner, LisbethBalão Da Silva, CarolinaMarouane, HafedhBalčiūnienė, JūratėMarrano, NicolaBalestrino, RobertaMarrone, Michael T.Balogh, ZsoltMartelli, FrancescoBandurska-Stankiewicz, ElżbietaMartí, JoaquínBär, SarahMartín-de-Llano, José JavierBaracchini, ClaudioMartinez Garcia, Miguel AngelBaran, MarzenaMartínez Sanz, José MiguelBarbieri, LaviniaMartínez-Åguila, AlejandroBarbu, LiviuMartínez-Arnau, Francisco MiguelBarcala-Furelos, RobertoMartínez-Ortega, Antonio JesúsBarnish, MaxMartingano, PaolaBarra, FabioMartín-Rodríguez, FranciscoBarrasa, HelenaMartins, FranciscoBarrios-Rodríguez, RocioMartucci, GennaroBarter, JolieMartynowicz, HelenaBartuzi, ZbigniewMaruya, KoheiBaskaran, RathinasamyMaruyama, TesshoBasoli, ValentinaMarzilli, MarioBassett, DarrylMasaaki, TakeuchiBathula, Chandra SekharMasiak, JolantaBatool, MariaMason, Howard J.Battista, MarcoMassa, ValentinaBaylina, PilarMassart, AlainBayoumi, AliMasuda, CamlynBazhanova, Elena DMasutani, KosukeBeales, Ian L.P.Masutani, MitsukoBean, Anthony M.Mateo, Daniel ColladoBechir, AnamariaMateus, SóniaBechsgaard, ThorMathew, MonaBednarska-Szczepaniak, KatarzynaMathews, RanjivBeggs, Clive B.Matsubara, ShigekiBegin, PhilippeMatsumoto, ShokeiBejarano, EloyMatsuura, ShinobuBellin, Marie FranceMatsuzaki, ShinyaBen Hammouda, ManelMatteo Biafora, MatteoBenatti, BeatriceMatucci-Cerinic, MarcoBenitez Porres, JavierMaugeri, Andrea GiuseppeBentley, DavidMaulucci, Christopher M.Benton, Angela H.Mauro, Adolfo GabrieleBergamaschi, RobertoMaurya, ShailendraBerger, Amnon A.Mavilio, AlbertoBerger, JoshuaMayer, PhilippBerking, CarolaMayer-Pickel, KarolineBernardazzi, ClaudioMayordomo, RaquelBernardi, SaraMayr, JohannesBernasconi, CorradoMazoteras-Pardo, VictoriaBernuy-Guevara, CoralinaMcArthur, CaroleBerteau, Jean-PhilippeMcGrath, Kathleen H.Bertelli, ElenaMcKeever, Kenneth H.Bertolotto, MicheleMcKenna, Peter EdwardBertomeu-Gonzalez, VicenteMcKinney, Matthew StuartBhat, AmbarishMcLachlan, StelaBhat, Ambarish P.McNarry, MelittaBhattu, Amit SatishMead, Matthew P.Bhogal, PervinderMeade, Maureen O.Bhutta, MahmoodMedina-Perez, CarlosBiagi, RobertoMeduri, AlessandroBiagioli, ValentinaMeesters, KevinBickel, ScottMehdi, EbrahimiBieleninik, ŁucjaMeima, Marcel E.Bieliński, MaciejMeinhardt, MatthiasBild, WaltherMelo, MariaBilger, AndreaMelrose, JamesBilski, JanMempin, MariaBiondi, AntonioMenahem, SolomonBirgin, EmrullahMendez, Mario F.Birjandi, Anahid AhmadiMercer-Chalmers-Bender, KatjaBirlea, MariusMercury, Santo RaffaeleBjorklund, AnneliMerk, DanielBlanco-Gandía, M.CarmenMero, AnttiBlumenberg, AdamMesker, Wilma E.Bob, FlaviuMessiah, AntoineBocchieri, SalvatoreMeszaros, MartinaBoczar, DanielMetere, AlessioBodendorfer, Blake M.Meyer, Till JasperBodenham, Andrew R.Michalak, EwaBoer, LucasMichálek, PavelBoerma, MarjanMigita, OhsukeBoger, HeatherMignano, AntoninoBonan, Paulo Rogério FerretiMihala, GaborBoniewska, EwaMikolajczyk, AgataBonini, SergioMillin, Paula M.Bonometti, ArturoMillman, Zachary BenBoon, ChengMills, Paul J.Bordonaro, MichaelMin, Hyun-jinBorghesi, AndreaMin, ZawBornstein, Natan M.Mindrila, DianaBorovac, Josip A.Minervini, GiuseppeBortoluzzi, CristianoMinghelli, BeatrizBorzage, MattMingoli, AndreaBoschetti, GiovanniMingot-Castellano, María EvaBossi, PaoloMinotti, BrunoBourdon, EmmanuelMiranda-Rius, JaumeBourel-Bonnet, LineMirijello, AntonioBoussios, StergiosMirjalili, AliBove, TizianaMishra, AbheepsaBover, JordiMishra, MuditBozek, AndrzejMishra, PradyumnaBozic, JoskoMitamura, TakashiBozkurt, SelimMitani, SeiichiroBozso, Sabin J.Mithani, KarimBraaf, SandraMitsea, AnastasiaBrady, Patrick G.Mitsea, Anastasia G.Bransfield, RobertMiwa, KunihisaBrasic, JamesMiyagawa, ChihoBraun, Frank K.Miyamura, SatoshiBravatà, ValentinaMiyoshi, TakekazuBreederveld, Roelf S.Mizia-Stec, KatarzynaBrindisi, MatteoMladenova, IrenaBroadhurst, C. LeighMladinescu, FiraBroggi, GiuseppeMoazed, FarzadBrogi, EtruscaMobasheri, AliBrogna, BarbaraMoccia, MarcelloBrogna, ClaudiaMöhlendick, BirteBrooks, John K.Mokadem, MohamadBrown, FreddyMolero, PatricioBrown, Ronald S.Moletta, LuciaBrowne, CarolineMolina-Cerrillo, JavierBrownell, PatriciaMomm, FelixBruggmann, PhilipMoniche, FranciscoBruno, VincenzoMontagnani, AndreaBruschini, LucaMontanino, MarioBrusini, PaoloMontemezzi, PietroBryndza, Magdalena A.Monzani, AliceBudd, JohnMoran, CarlaBullens, Dominique M.A.Moratin, JuliusBuonsenso, DaniloMoreo, GiuliaBur, AndresMorgan, Jessica I.W.Burghardt, KyleMorgan-Bathke, MariaBurgos-Ramos, EmmaMorin, MathieuBurucoa, ChristopheMorin, MatiasBusana, MattiaMorio, YoshiteruBuselli, RodolfoMorita, KyojiBusin, MassimoMoszynski, RafalBustos, RobertoMotamed, CyrusBuus, Niels HenrikMoulis, GuillaumeByl, Nancy NMourão, Carlos FernandoBystrom, JonasMoxham, BernardByun, JinyoungMoya-Jiménez, María JoséCabaton, JulienMuhammad, NaoshadCaccianiga, GianluigiMuhvic Urek, MirandaCadranel, Jean François D.Muiesan, Maria LorenzaCaffarelli, CarlaMukerji, MitaliCafolla, ArturoMukherjee, NabanitaCalda, PavelMukherjee, RatnadeepCalkins, HughMüller, GünterCalò, Pietro GiorgioMuller, SaraCalvo Lobo, CésarMüller, ThomasCamargo, Samira Esteves AfonsoMüller, WolframCameron, DanielMumcuoglu, Kosta Y.Cameselle-Teijeiro, Jose ManuelMumoli, NicolaCampanacci, LauraMunday, MichaelCampanati, AnnaMunyaka, Peris M.Campanella, ClaudiaMünzer, PatrickCampanile, Fabio CesareMurakami, KotaroCampbell, KatarzynaMurakami, YoshikiCampello, ElenaMurawski, BeatriceCampi, IreneMurray Stewart, TracyCampioni, IlariaMusani, VesnaCañadas-De La Fuente, Guillermo A.Mushtaq, NasirCanakoglu, ArifMusiello, Rubens BermudesCanavese, FedericoMusk, BillCândido Carvalho, KátiaMustachio, LisaCangiano, BiagioMusumeci, GiuseppeCannarella, RossellaMyasoedova, VeronikaCannavò, Serafinella P.Myers, Stephen DavidCannella, RobertoMynampati Arunadithya, Bharani KrishnaCapocasale, GiorgiaMyrkos, AristeidisCaponio, Vito Carlo AlbertoMyśliwiec, PiotrCaravale, BarbaraNadisauskiene, RutaCardona, VictoriaNagarajan, PrabakaranCarella, Angelo MNaghibalhossaini, FakhraddinCarelli, StephanaNagwekar, JanhaviCarlomagno, ChiaraNaito, YutakaCaron, RosemaryNakagawa, MayumiCarotenuto, MarcoNakai, MichikazuCarotti, SimoneNakai, YousukeCarrilho, EuniceNakajima, TakahiroCartwright, TinaNakanishi, MiharuCaruso, FrancescoNakatani, YosukeCaruso, SalvatoreNaldi, LuigiCasado, ArturoNam, Ok HyungCasanova, Alfredo GinésNanchahal, JagdeepCasciaro, MarcoNapolitano, RobertaCassady, Steven J.Narayanan, SankaranCastellano, OrlandoNarkhede, MayurCastilletti, ConcettaNascimento, HenriqueCastleberry, ToddNasry, WalaaCastro Alves, RicardoNath, ShubhankarCastro-Codesal, Maria LuisaNault, Marie-LyneCatone, GennaroNavarro Flores, EmmanuelCavagnetto, DavideNawas, Afshan FathimaCaziuc, AlexandraNayak, Manasa K.Cebríán-Carretero, José LuisNaydenov, StefanCeccarelli, GabrieleNeculau, Andrea ElenaCedars, AriNeeb, AntjeCejudo, AntonioNekludov, MichaelCensi, SimonaNemeș, Roxana MariaCerbone, LuigiNeshatian, LeilaCernadas, JosefinaNesic, DobrilaCerník, DavidNeuner, BrunoCerrone, MargheritaNewson, LisaCervera-Garvi, PabloNexø, EbbaChacón, EnriqueNicolas-Silvente, Ana IsabelChadman, KathrynNiebel, DennisChakraborty, JayasreeNiemira, MagdalenaChakravarty, ShatadruNieto Fontarigo, Juan JoseChalah, Moussa AntoineNifli, Artemissia-PhoebeChandrasekharan, SankaranNiikura, RyotaChang, Jia-FengNikolic, DejanChang, Ke-VinNishikawa, JunChang, Ling-ChuNishikawa, TetsuoChang, Nai-JenNordio, MaurizioChanoine, Jean-PierreNorihiko, NaritaChao, Chia-TerNourazari, SaraChappell, Mark C.Nouri, AriaCharmas, MalgorzataNowaczyk, SławomirChatzikyrkou, ChristosNowak, Michal S.Chaushu, GavrielNowakowska-Lipiec, KatarzynaChecchi, VittorioNowomiejska, KatarzynaCheemarla, NagarjunaNucera, RiccardoCheemarla, Nagarjuna ReddyNunamaker, ElizabethChen, Der-YuanNúñez-Iglesias, María JesúsChen, GuanminO’ Connor, DominicChen, HuO’Connor, Elizabeth A.Chen, Kenneth K.O’Neal, EricChen, Tai-HengO’Sullivan, CormacChen, XiaoyanOakey, ZackeryChen, YangOberkircher, LudwigChen, Yi-LangOda, YoichiroChen, YingOdintsova, VeronikaCheng, Fu-JenOgawa, AsaoCheng, Kai-ChunOgonowska, AnnaCheng, Pi ChunOhara, YukohChepelev, LeonidOhki, MasafumiCheung, AdaOhko, KentaroChien, Lung-changOhtsuka, TakashiChimen, MyriamOjetti, VeronicaChimenti, RuthOkano, IchiroChinnasamy, AlagesanOkuda, KazuhideChintalapudi, SumanaOkumura, TakahiroChioncel, OvidiuOliva, StefaniaChirico, FrancescoOliveira, Mariana Bleker DeChiu, Chia-YuOliver, DavidChoi, DonghoonOlshansky, BrianChoi, June YoungOlszewska, MartaChoi, MyeongjinOltra, ElisaChoi, Won SukOmodior, OghenekaroChroni, ElisabethOng, JohnChuah, Alice Lay HongOnisor, FlorinChuang, Hung-YiOnișor-Gligor, FlorinChung, Felicia Fei-LeiOostra, Roelof JanChung, Ki WhaOrand, AbbasChung, Yong AnOranges, TeresaChurchwell, MariannOrlacchio, ArturoCianchetti, CarloOrtega, Joseph ThomasCiarambino, TizianaOrtolano, SaidaCici, DanielaOtani, HidenoriCiclitira, PaulOtsuki, BungoCieślik, BłażejOwczarek, Aleksander JerzyCigna, EmanueleOwen, Patrick J.Cillino, GiovanniOyen, RaymondClark, Nicolas W.Paetzold, BernhardClark, ToshiPafundi, Pia ClaraClarke, SusanPaganini, MatteoClaus, Ralf A.Pagotto, SaraClemente-Suárez, Vicente JavierPaireder, MatthiasClemente-Suarez, Vincente JavierPal Choudhuri, ShreoshiClevenger, KimberlyPaladino, Nunzia CinziaCoates, LauraPalatyńska-Ulatowska, AleksandraCoccaro, NicolettaPaleg, VirginiaCochran, AllysonPalini, SimoneCoffey, NiamhPallesen, StåleCohen, DanielPalmer, AlanColeman, CynthiaPalmiero, GiuseppeColizzi, MarcoPalmu, Sauli ACollado-Mateo, DanielPanagiotaki, GeorgiaColosi, Horațiu AlexandruPanagiotidou, EvangeliaComi, GiancarloPandey, RajeshConde, BebianaPandhi, AbhiCondor, Jose ManuelPane, KatiaConfalonieri, MarcoPanicker, VijayConnolly, Mark P.Panidi, IoliConserve, Donaldson F.Panigrahy, DipakConstantinescu-Aruxandei, DianaPanos, Anthony L.Constantinou, Costas S.Panza, RaffaellaConte, MassimoPaolino, GiovanniCook, James L.Papachristodoulou, AlexandrosCoppedè, FabioPapachristos, Ioannis V.Cordeanu, Elena-MihaelaPapadopoulos, Elias G.Córdoba, OctaviPapanagiotou, PanagiotisCornelis, FrancoisPapp, NoraCornish, StephenPaprocka, JustynaCorradin, MarcoParagliola, Rosa MariaCorral Varela, MontserratParanjape, AnuragCortese, AntonioPark, Joon YoungCosmi, EricPark, Jun-BeomCostanzo, LucaPark, Jun-ookCostello, Kerry E.Park, Ki-cheongCouvy-Duchesne, BaptistePark, Kwan-KyuCox, Linda S.Parlatescu, IoaninaCozar, IParry, SionCozzolino, ImmacolataPassarelli, Pier CarmineCraig, Bruce W.Pastor, ZlatkoCrawford, KimberleyPaszynska, ElzbietaCriado-Alvarez, Juan JoséPatel, UrvishCrincoli, VitoPatelarou, AthinaCristofaro, Raimondo DePateras, Ioannis S.Croagh, DanielPaterson, Rebecca S.Cronkite, Ruth C.Patini, RomeoCrosio, AlessandroPatriat, RemiCsincsik, LajosPattappa, GirishCui, QingbinPatwardhan, Avinash R.Culler, CorinnaPavez Lorie, ElizabethCulver, Daniel A.Pavol, Marykay A.Cunha Rudge, Marilza VieiraPawliczak, RafałCuric, GoranPayrastre, BernardCzyż, KatarzynaPaz Montelongo, SorayaD. Bortner, CarlPečiulienė, VytautėD’Alonzo, DanielePeixoto, EstanislaoD’Angelo, AlbertoPellesi, LanfrancoD’Arrigo, SoniaPellicano, RinaldoD’Orazi, ValerioPelliccioni, Gian AndreaDa Silva, IvanPeloso, AndreaDabrowski, FilipPeng, MeiDąbrowski, WojciechPennicooke, BrentonDacrema, MarcoPenumarti, AnushaDadoniene, JolantaPercudani, Mauro EmilioDagklis, Themistoklis I.Perenc, LidiaDahl, Jon E.Peres, Bernardo U.Dambrova, MaijaPérez-Herrero, María A.Danielewicz, AnnaPeris, LolaDanso, SamuelPerkins, Adam M.Dar, Wasim A.Perricos, AlexandraDas, Bibhuti BhusanPerrier, VeroniqueDascalu, MariaPerrotta, AdolfoDassieu, LisePerry, LeslieDaugule, IlvaPetca, RǎzvanDaunoraviciene, KristinaPeters, CaitlinDave, SandeepPetersen, SvenDavid, Vlad LaurentiuPetkari, EleniDavidow, AmyPetráš, MarekDavis, Mellar P.Petrikaitė, VilmaDay, KaitlinPetritsch, BernhardDe Angelis, PaoloPetrovan, VladDe Beule, PieterPetruzzelli, Maria GiuseppinaDe Cock, DiederikPeyrony, OlivierDe Fazio, PasqualePezzilli, RaffaeleDe Francesco, FrancescoPfaff, JohannesDe Hoz, RosaPheby, DerekDe Julián Ortiz, JesusPhilipp, AlexanderDe Lellis, LauraPhojanakong, PamDe Palma, GiadaPhulera, SwastikDe Pastena, MatteoPiacenti, IlariaDe Ridder, DirkPiagkou, MariaDe Rosa, VivianaPiccin, AndreaDe Sire, RobertoPichiecchio, AnnaDe Sousa-Coelho, Ana LuísaPiciu, DoinaDe Stefani, AlbertoPié, JuanDe Vito, ElisabettaPietrzak, BronislawaDe Vroege, LarsPilic, LetaDeb, SiddharthaPilutin, AnnaDeb, SubrataPinhas-Hamiel, OritDecker, Brooke K.Pinheiro, Joaquim M. B.DeCoursey, ThomasPintye, JillianDeftereos, Spyridon G.Pipolo, CarlottaDe-la-Cruz-Torres, BlancaPippias, MariaDelitala, AlessandroPirani, VittorioDellavia, ClaudiaPironi, DanieleDelles, ChristianPirtea, LaurentiuDelmonico, Matthew J.Pitarch, AidaDelprat, BenjaminPitti, Julio AlvarezDelussi, MariannaPlaat, Boudewijn E. C.DeMartini, JuliePlayford, Raymond JohnDemer, Joseph L.Pleass, HenryDemetrovics, ZsoltPodfigurna, AgnieszkaDenis, FredericPolańczyk, AndrzejDentino, Andrew R.Polastri, MassimilianoDepalma, Ralph G.Poli, AlessandroDesai, MoreshwarPolissidis, AlexiaDesJardins, John D.Politynska, BarbaraDeStefano, Christin B.Pollesello, PieroDevy, ZismanPolz-Dacewicz, MałgorzataDewar, RajanPonce-Blandón, José AntonioDexheimer, JoshuaPonikau, Jens U.Di Diego, JosePop, Laura AncuţaDi Fiore, AdolfoPop, Tudor LucianDi Gianfilippo, RiccardoPopa, Daniela-SavetaDi Prete, MoniaPopescu, Iulia DanaDi Spirito, FedericaPopescu, Sanda MihaelaDi Vincenzo, AngeloPorela, PekkaDiac, Mădălina MariaPoškus, EligijusDiaconu, CameliaPotaczek, Daniel P.Díaz-Jiménez, José PabloPott, AlexanderDickinson, Dale A.Pourafkari, LeiliDidilescu, AndreeaPoznanski, PiotrDietch, Jessica R.Pozo Sánchez, SantiagoDiez, YagoPreckel, KatrinDigby-Bowl, Caroline J.Principia, Scavo MariaDilger, James P.Printza, NikoletaDimienescu, Oana GabrielaPriya, SarvDimopoulos, KonstantinosPrnjak, KatarinaDipchand, ChristineProcentese, FortunaDiserens, KarinProchor, PiotrDIVAKARUNI, SRINIVASPrudhon, Jean-LouisDixon, Anthony J.Puga, Ana M.Djordjevic, IlijaPugliese, PierfrancescoDkhar, Hedwin KitdorlangPulle, Ranjeev ChrysanthDobersek, UrskaPurdy, MikeDocimo, LudovicoPuri, KritiDodoo, ChristopherPursey, KirrillyDoi, HisashiPuślecki, MateuszDolezel, Diane MQasim, MuhammadDoll, Margaret K.Qiao, AijunDomanin, MaurizioQureshi, FawadDomingo, MarRachele, BrancaleoniDomingo-Musibay, EvidioRadzevičienė, AurelijaDommerholt, JanRaffa, GiovanniDonadello, KatiaRaimondo, Domenico DiDonaldson, AlexRaj, Isaac SelvaDonati, MarcelloRamírez Soto, Max CarlosDonkers, SarahRamirez, Jose ManuelDono, JoanneRamirez-Graniz, Irwin AndrésDontukurthy, SujanaRamprasath, TharmarajanDookeran, Keith A.Ramsay, Michael A.Dosil, MariaRanjan, PeeyushDott, Giovanni MarascoRao, Rammohan V.Douda, TomášRapado-González, ÓscarDouglas, VanjaRaposio, EdoardoDoulberis, MichaelRatwani, RajDragonieri, SilvanoRau, BeateDream, SophieRau, IleanaDu, HaishunRav Acha, MosheDubar, MarieRawla, PrashanthDubinin, Mikhail V.Raziel, AsnatDuda, Marcel MateiRecalde, SergioDudzińska, EwaReece, Edward M.Dullenkopf, AlexanderReed, Amy McCartDumler, FrancisRefolo, GiuliaDunne, Colum P.Refoyo, IgnacioDuployez, NicolasRehman, ShehzadDusi, VeronicaReichenbach, Zachary WilmerDuvekot, Johannes J.Remmenga, Steven W.Dziedzic, ArkadiuszRengasamy, ManivelEadie, LauraRengo, MarcoEbo, DidierRenke, JoannaEbraheim, NabilRenom, Josep Maria SerraEddie, DavidReuther, Katherine E.Edelman, David A.Revelli, LucaEdwards, VinceReychler, HervéEgelund, Eric FreeRiad, AbanoubEhler, JohannesRibeiro, PaulaEhrentraut, Stefan FelixRicciarelli, RobertaEhrmann, MichaelRichards, Dylan JackEibenschutz, LauraRichter, JanEisenmann, StephanRichter, SusanEkúndayò, Olúgbémiga T.Richter-Dahlfors, AgnetaEl Mobadder, MarwanRicou, MiguelElessawy, MohamedRider, CynthiaEl-Sheikh Ali, HossamRiedel, FabianEngström, NiklasRiess, HannoEnninga, Elizabeth Ann L.Rieth, Andreas J.Ercia, AngeloRigante, DonatoEreminienė, EglėRigby, Matthew H.Ersen, AliRimaitis, KęstutisEscallon, JaimeRingdén, OlleEscuriet, RamonRivero-Cruz, J. FaustoEsfandyari, SaharRizos, HelenEstomba, Carlos Miguel ChiesaRobb, Nigel DEstravís, MiguelRoberts, Victoria H. J.Eto, KojiRobin, Jérôme D.Ettcheto, MirenRobins, Jo Lynne W.Ettenberger, MarkRoceanu, AdinaEu, PeterRoche, FrédéricEun, Ki-SooRochfort, KeithFagerstrom, KarlRodilla, EnriqueFaitot, FrancoisRodrigo, LuisFalk, MartinRodrigo, MiguelFalsaperla, RaffaeleRodriguez, LuisFalup-Pecurariu, OanaRodriguéz, Olga CatalinaFan, Hueng-ChuenRodríguez-Rubio, M.Fansa, HishamRoggio, FedericoFantozzi, Paolo J.Roje, DamirFaravelli, IreneRolla, GiovanniFarhan, SerdarRomero-Gómez, BenjamínFarokhi, Moshtagh R.Rommel, FelixFavaloro, EmmanuelRoncal, CarmenFavara, GiulianaRoncero, CarlosFeatherstone, John DRosa, DeboraFederico, De Montalvo JaaskelainenRosenberg, MaiFedosov, SergeyRosenberg, NahumFerguson, BradleyRoskar, SaskaFernandez, Manuel NunezRosołek, BarbaraFerrara, MariaRoss, RyanFerrara, ZacharyRosser, Tena L.Ferrari, FedericoRossi, AlessioFerrone, FedericaRossi, TaniaFidler Mis, NatasaRostoker, GuyFillmore, PaulRoth, DominikFine, NoahRovas, AlexandrosFinocchi, CinziaRove, Kyle O.Fiorillo, AndreaRozerta, SokouFiorillo, LucaRozsival, PavelFirmino, HoracioRubin, MosheFischer, BastianRübsamen, NicoleFischer, Nicholas G.Rudin, DeborahFischetti, FrancescoRudrappa, MohanFisk, Malcolm J.Ruggeri, Rosaria M.Flaherty, KevinRuggiero, AngeloFleischer, Arthur C.Rugytė, DanguolėFlessas, IoannisRumba-Rozenfelde, IngrīdaFliefel, Riham M.Ruppert, KristineFlorens, NansRush, Eric T.Foerster, KatharinaRussell, DanielFolegatti, PedroRusso, RiccardoFoletto, MirtoRutegård, MartinFolgosa, FilipeRutz, ErichFonseca, Elsa S.R.Ryozawa, ShomeiForbes, AlastairRyu, Mi-HeonForest, FabienSaad, MarceloForget, PatriceSaar, MatthiasForjaz, ClaudiaSabaliauskaitė, RasaFormanowicz, DorotaSabarimurugan, ShanthiForte, GiuseppeSabbatini, SamueleFortunato, AlexandroSabia, MichaelFoster, Kenneth R.Sacchini, VirgilioFotiou, DespinaSacco, EmilioFragomeni, GionataSacristán, José AntonioFrancesco, GrandoniSadasivam, MohanrajFrazzoni, LeonardoSadrin, StéphaneFredsøe, JacobSæbø, GunnarFreitas, TomásSaeedi, PouyaFriedler, ShevachSaha, RenataFriel, Ciarán P.Sahinovic, Marko M.Frohnhofen, HelmutSaibene, Alberto MariaFrolíková, MichaelaSak, Jarosław J.Frucht, Steven J.Sakai, SatoshiFu, Pin-KueiSakamoto, ShinichiFuhrmann, ChristopherSakurada, KazuhiroFujibe, FumiakiSala, RobertoFujimura, ShintaroSałaga, MaciejFujioka-Kobayashi, MasakoSaláková, MartinaFujita, KazutoshiSalgado-Castro, Francisco JavierFukutani, AtsukiSalomó-Coll, OscarFulda, Kimberly G.Salvado, FranciscoFunel, NiccolaSalvati, BrunoFurutani, KentaSalzano, EmanuelaFusar-Poli, LauraSam, RaminGabryś, TomaszSammartino, PaoloGacek, MariaSampedro, PatriciaGaier, EricSampogna, GaiaGajdzis, PawelSánchez-Bueno, FranciscoGALANIS, NikiforosSánchez-Gómez, María BegoñaGalasso, DomenicoSánchez-Sanchez, ManuelGallo, GaetanoSands, Jeff M.Galvagno, Samuel M.Săndulescu, MihaiGambardella, ClaudioSani, GabrieleGaragiola, UmbertoSantarelli, AndreaGarcía, Ana M.Santulli, GaetanoGarcía, José RamónSanzo, AntonioGarcia, SergioSanz-Paris, AlejandroGarcia, VictorSapalidis, KonstantinosGarcía-Davis, SaraSarabia, Jose ManuelGårevik, NinaSarani, BabakGaribotto, GiacomoSarshar, MeysamGaridkhuu, AriuntuulSasaki, HirakuGarrett, Mario D.Saul, DominikGarrote-Adrados, José AntonioSaunders, AnnetteGartner, Lawrence M.Savic, SinisaGarus-Pakowska, AnnaScalinci, Sergio ZaccariaGarzon, SimoneSchara, UlrikeGarzon, XimenaSchenkel, Lindsay S.Gatz, MatthiasSchiattarella, AntonioGatzoulis, Konstantinos A.Schinke, ThorstenGawel, KingaSchlegel, JürgenGazal, GiathSchleip, RobertGeerts, DirkSchmutz, AxelGemmill, AlisonSchoon, YvonneGenesio, RitaSchuldt, TobiasGenovesi, DomenicoSchultz, StephenGeorge, KoumantakisSchurink, BernadetteGeorgian, BadicuSchweyen, RamonaGeorgiev-Hristov, TihomirScicchitano, PietroGerding, HeinrichScuderi, VincenzoGerhart, HaydenSeaby, EleanorGerlach, HerwigSebastianelli, ArcangeloGervais, NicoleSeetharaman, MahadevanGesesew, Hailay AbrhaSegers, SeppeGhidini, MicheleSekiguchi, YasukiGhilli, MatteoSemedo, Carla SantarémGhimire, KedarSeo, BenedictGhione, PaolaSeo, HyungseokGhosh, SouravSerban, Daniela ElenaGiacomo, LazzeriSerban, DragosGiamundo, PaoloSertie, Rogerio Antonio LauratoGiangregorio, FrancescoSeth, AnushreeGiannì, MariaSetsuya, AibaGianno, FrancescaSevastianos, VassiliosGianoncelli, LetiziaShamah, Steven P.Giles, GregoryShapiro, Daniel E.Gill, TiffanySharma, GauravGillet, PierreSharma, HariGiménez, NuriaShekargoftar, MasoudGiorgi, Alfredo DeShemyakin, ArkadyGiovanni, MerlinoShen, MengchengGiovingo, Michael CarloShen, XingguiGiri, HemantSheng, YueGiuffrè, MauroShepherd, Charles C.Giuseppe, VarvaraShepherd, S.Gleiznienė, RymantėSheppard, Mary N.Glenister, KristenShiao, Chih-ChungGlinkowski, WojciechShibagaki, KotaroGlover, Anthony R.Shin, Jae IlGlowniak, AndrzejShiraishi, IsaoGobba, Fabrizio MariaShivakumar, PranavGodlewski, GrzegorzSiddle, Heidi J.Goel, RahulSidiropoulos, KonstantinosGogulamudi, Venkateswara ReddySignori, EmanuelaGołębiewski, MarcinSilva, Andreia M.Goll, RasmusSilva, Diego Marconi Pinheiro FerreiraGonon, GeraldineSilva, Rona M.González, JerónimoSilvestre-Rangil, JavierGonzalez, MichelSilveyra, PatriciaGonzález-Bulnes, AntonioSimonelli-Muñoz, Agustín JavierGonzález-García, CristinaSimoni, NicolaGonzález-García, HiginioSin, AnthonyGonzález-López, Tomás JoséSinger, JosefGonzález-Martín, OscarSingh, Anand PrakashGonzález-Mesa, ErnestoSingh, AnupGonzalez-Pujana, AinhoaSingh, ParamGonzalez-Suarez, Maria L.Singh, RaveshGorgulu, KivancSingh-Bains, Malvindar K.Gorini, AlessandraSinghvi, AditiGorini, FrancescaSinha, DebottamGörlich, DennisSinjab, KhaledGos, ElżbietaSinnberg, Tobias W.Gosav, Evelina MariaSinnott, Catherine J.Granados-Principal, SergioSirinian, ChaidoGranata, AntonioSisask, MerikeGrandjean, Peter W.Sisti, DavideGranieri, EnricoSisu, CristinaGrasby, KatrinaSjöqvist, SebastianGray, BenSkarzynski, Piotr HenrykGraziani, FilippoSkórzyńska-Dziduszko, KatarzynaGreaney, HilarySkride, AndrisGribsholt, Sigrid BjergeSkrobot, WojciechGrimm, MarcusSkrzypiec, GraceGrjibovski, AndrejSlezák, RadovanGromov, IrinaSłomka, ArturGrosicki, GregSłomko, JoannaGrotta, James C.Smahel, MichalGrubić Kezele, TanjaSmall, Roy S.Grune, TilmanSmietana, MarcinGrygorowicz, MonikaSmolarczyk, RomanGrzegrzółka, JȩdrzejSnowdon, David A.Gu, YuchaoSobrido-Cameán, DanielGuadalajara-Labajo, HectorSocea, BogdanGuaragna, AnnalisaSöhling, NicolasGubbels, Ashley L.Solanki, SumeetGudey, Shyam KumarSolarino, GiuseppeGuertler, AnneSolimando, Antonio GiovanniGugel, IsabelSolís García Del Pozo, JuliánGuidoin, RobertSommantico, MassimilianoGuillen-Sola, AnnaSon, KeunBaDaGuirguis, AmiraSonavane, ManojGun, RichardSoncini, MarcoGunasekar, Susheel K.Song, Kwang-SupGunning III, William T.Soong, WenjueGuo, XiufangSophocleous, FrosoGuppy, FergusSørensen, SuzetteGupta, NehalSoto-Hermida, AngelGupta, VikasSottosanti, EmilyGurupur, Varadraj PrabhuSourial, Maryanne Y.Gustad, Lise TusetSouza-Dantas, Vicente CésGuzzardi, GiuseppeSpang, ChristophHaase, Elaine M.Spartalis, EleftheriosHaber, Lynne T.Spaulding, Aaron C.Hachisu, YoshimasaSpeer, Esther MonikaHackett, DanielSpeight, NigelHadigal, SusheelaSpeth, BernhardHadjimbei, ElenaSpinelli, LetiziaHadkhale, KishorSpivakovsky, SilviaHaese, Nicole N.Sporea, IoanHakovirta, Harri H.Sprave, TanjaHalaris, AngelosSpringer, SvenjaHale, BeverleySprong, Matthew EvanHall, Emily J.Sram, RadimHalsall, David J.Srejovic, IvanHalvachizadeh, SaschaSrinivasan, BharaniHamaoka, TakutoSrinivasan, Bharani G.Hammer, RichardStaderini, EdoardoHan, YuyanStančiaková, LuciaHanisch, MarcelStanciu, Luminita A.Hanne, NicholasStanescu, Ana Maria AlexandraHansson, ErikStanhope, JessicaHarada, YukinoriStanley, Jone A.Harel, RanStark, Amy L.Harper, AlanStark, MichaelHarris, EmmaStaudacher, Jonas JaromirHarris, Randall E.Stay, SharonHartmann, DanielaStefanska, AnnaHarty, PatrickSteiger, StefanieHascup, KevinSteinvall, IngridHasegawa, HiromasaStellavato, AntoniettaHasegawa, YukihiroStephan, DominiqueHashemian, MaryamStieber, JulianeHashimoto, YoshitakaStöcker, FabianHasoon, Jamal JamesStraccia, PatriziaHassan, AmanyStröhle, MathiasHatano, YuichiroStupica, DašaHavre, Roald FleslandSu, ChangqingHayes, RachelSuciu, OanaHazen, AlexesSuga, TakayukiHeaton, Steven M.Sukegawa, ShintaroHeinemann, FabianSullivan, Lori S.Helenius, IlkkaSumner, WaltonHelliwell, PhilipSun, WeinaHempel, GuntherSuomi, ReimaHemprich, AlexanderSuriano, FrancescoHentrich, Marcus U.Suzuki, AyakoHermida Prieto, ManuelSwami, UmangHerrero, Azael J.Swank, Douglas M.Herrick, Linda M.Sweiss, NaderaHessheimer, Amelia J.Świątkiewicz, IwonaHewer, WalterSyed, Taseen AhmedHewitt, JonathanSyed-Abdul, Majid MufaqamHey, JeremiasSzaflarska-Popławska, AnnaHeyman, Joanne K.Szczepankiewicz, AleksandraHeyse, AlexSzékely, AndreaHibi, MasanobuSzkaradkiewicz, AndrzejHigaki, AkinoriSzulińska, MonikaHildreth, BlakeSzumilewicz, AnnaHildreth, Blake E.Szylińska, AleksandraHilhorst, MarcTabor, LaurenHirano, TsunahikoTae, Hyun-JinHisako, YoshidaTagliapietra, MatteoHite, Robert DuncanTajika, MasahiroHo, VincentTakahashi, ToshifumiHogarth, LukeTakamata, AkiraHogg, JenniferTakami, AkiyoshiHöhn, AnnikaTakaya, JunjiHolcman, KatarzynaTakebayashi, ShigeoHo-Le, Thao P.Takeuchi, RikiyaHolland-Cunz, StefanTam, Jonathan S.Hong, Chien-HuiTampa, MirceaHopp, RussellTamura, ShogoHopp, Russell JamesTan, LeeHorowitz, JamesTanaka, HiroshiHosoyamada, MakotoTanaka, Hiroyoshi Y.Hotta, RyoTanaka, Kenichi A.Houben-Wilke, SarahTanaka, YutoHouvenaeghel, G.Tang, Paul C.Howe, CherylTanno, Luciana KaseHsiao, Sheng-MouTarantino, GiovanniHsu, Chia-ChunTarazona-Santabalbina, Francisco JoséHu, BoTargarona, EduardoHuang, Hui-ChunTarleton, Emily K.Huang, XiaohuTashani, Osama A.Hubková, BeátaTate, WarrenHuchzermeyer, CordTattoli, LuciaHughes, TimothyTatullo, MarcoHwang, Dae-seokTeixeira, LaetitiaIatrakis, GeorgiosTelyshev, DmitryIba, ToshiakiTentes, Antonios-Apostolos K.Ichibori, YasuhiroTeper, SlawomirIchkova, AleksandraTerranegra, AnnalisaIde, KazukiTerzella, Michael J.Ierardi, EnzoThavamani, AravindIglesias-Osma, Maria CarmenTheilmann, LorenzIgnatowicz, AgnieszkaThen, CorneliaIgo, RobertThirunavukkarasu, ShyamalaIgoe, AnnThivel, DavidIijima, ShigeoThomas, Anusha ShirwaikarIino, ChikaraThornton, KarleenIkeda, KeigoTian, Kun VivianaImafuku, ShinichiTienari, PenttiImai, NaohikoTikhonova, SvetlanaInchingolo, FrancescoTizek, LindaInglada-Pérez, LucíaTobyn, GraemeInns, StephenTocaciu, ShreyaInoki, KazuyaToffoli, AndreaIntagliata, SebastianoTofolean, Doina EcaterinaInyushin, Mikhail Y.Togawa, YaeiIolascon, GiovanniTokarek, TomaszIorga, AndreaTokodai, KazuakiIorio, RaffaeleTokuhashi, YasuakiIqbal, M. AnwarTomczak, AndrzejIrisawa, HiroshiTomescu, Mirela CleopatraIrusta, UnaiTong, JessicaIshiguchi, HironoriTonni, GabrieleIshii, HidekiTonon, MartaIsmail-Beigi, FaramarzTorisu, HiroyukiIsola, GaetanoTorng, Pao-LingItri, AngeloTorri, EmanueleIvarsson, BodilTorti, CarloIwaki, MichihiroToto, LisaIwamoto, ShotaroTotura, AllisonJackowiak, HannaToyokawa, TatsuyaJadlowiec, CarolineToyomasu, YoshitakaJagielski, MateuszTrabace, LuigiaJaiswal, Ashvin R.Trajkovski, BrankoJakimovski, DejanTrapani, DarioJames, Claire DTraynelis, Vincent C.Jang, SiwonTreuter, EckardtJanikowska, GrażynaTreviño, Claudia L.Janjua, Muhammad BurhanTriantafillidis, John KJankauskiené, AugustinaTriposkiadis, FilipposJanssens, ThomasTrivli, AlexandraJanuszek, RafałTroadec, Marie-BérengèreJasiewicz, BarbaraTrohman, Richard G.Jaskiewicz, MaciejTruszczyńska, AleksandraJatužis, DaliusTsai, Ming-ShaoJavier Jiménez-Jiménez, FelixTsai, Sandra A.Jayaraman, JayakumarTsai, SenweiJenkins, RachelTsakiridis, IoannisJeon, IkchanTsampras, NikolaosJeong, Keun-YeongTsaousis, Konstantinos T.Jesenak, MilosTsatsoulis, AgathoclesJezierska-Wozniak, KatarzynaTsoutsi, VagioulaJi, Yisi D.Tsuchiya, MasahikoJiang, ChunjieTsuda, HitoshiJillella, DineshTsukamoto, OsamuJin, LiTsukushi, SatoshiJoachim, MikelTsurumaki, HiroakiJochmanova, IvanaTucker, LarryJochum, ChristophTudoran, MarianaJohnson, DanielTundo, AntonioJohnson, SamTural, ÜmitJones, ArwelTurbanti, SimonaJones, PeterTuriel, MaurizioJonnalagadda, Anil KumarTurillazzi, EmanuelaJoosten, AlexandreTurkalj, MirjanaJoumma, VenusTurki, HoucemeddineJue, Joshua S.Turkkahraman, Muhammet HakanJuengst, Shannon B.Turnbull, Christopher DavidJuncar, MihaiTurner, Alice M.Jung, Se YongTurowski, GittaJungsuwadee, PaiboonTursi, AntonioKachamakova-Trojanowska, NeliTuvdendorj, DemidmaaKachrimanidou, MelinaTward, Daniel J.Kada, SundaranTzaridis, SimoneKagabu, MasahiroUeberschär, OlafKaissi, Ali AlUher, JanaKakkassery, VinodhUnger, EricaKalampokas, TheodorosUpadhyay, Kiran K.Kalasauskas, DariusUpadhyaya, JasbirKalaszczyńska, IlonaUrbanska, Ewa M.Kämmerer, Peer W.Urra, José MiguelKanaujiya, JitendraUrrialde De Andrés, RafaelKanduri, Swetha R.Usuda, HarukiKang, JeonghyunVaira, Luigi AngeloKang, Seung-BaikVaitkevičienė, GodaKanno, ToruValente, Nicola AlbertoKantola, Ilkka M.Valente, SabrinaKaoutzanis, ChristodoulosValentinčič, Nataša VidovičKaplan, ShaunaVali, PayamKapritsou, MariaVan Der Putten, Gert-JanKaraduta, OlegVan Egmond, LieveKarakatsani, DespoinaVan Regenmortel, NielsKaran M, ShahVan Slycke, SamKaratzas, PantelisVAN ZWIETEN, Koos JaapKarhula, SakariVancheri, FedericoKarim, Helmet T.VandenBussche, Christopher J.Karnati, SreenivasVanni, DanieleKarras, PanagiotisVargas, Sara OakesKarst, MatthiasVarvarousis, DimitriosKase, SatoruVasavada, BhavinKaskel, FrederickVasudevan, AbhinavKasprzak, JaroslawVaughan, BrettKassassir, HassanVelasco, Carlos E.Katz, JosephVelikova, TsvetelinaKawaguchi, AtsushiVellipuram, Anantha R.Kawaguchi, NanakoVellone, Valerio GaetanoKazachkov, MikhailVelotti, NunzioKędzierska, EwaVender, RonaldKeenan, Jeffrey A.Venditto, VincentKeller, M. JeanVeneri, Diana A.Kellett, John G.Venugopal, NiranjanKelly, PatrickVeras, MirellaKennedy, DavidVerde, FedericoKent, BrianneVerguts, JasperKhade, Natasha B.Verma, NupurKhandakar, BinnyVernero, MartaKhandelwal, SoniVeroux, MassimilianoKhedr, SherifVersacci, PaoloKheyfets, Vitaly O.Vervoort, GeraldKholová, IvanaVidetta, WalterKhoo, ClarenceVij, HiteshKhosrawipour, TanjaVijayakumar, SarathKhullar, SachinVikingsson, SvanteKhushwant Singh, BhullarVingolo, Enzo MariaKiat, HosenVirgolino, AnaKiciński, PawełViscido, AngeloKido, Mizuho A.Visconti, FedericaKikuchi, AtsuoVita, RobertoKikuyama, MasatakaVittori, AlessandroKilaikode, SasikumarVocaturo, EugenioKim, Gi BeomVoigt, Jens-UweKim, Ha-NeuiVolgman, Annabelle SantosKim, Ji HeuiVon Ameln-Mayerhofer, AndreasKim, Jin-WooVoon, Dominic Chih ChengKim, Kyeong OkVoros, DionysiosKim, Min YoungVyatkina, Kira V.Kim, Nam KyuWagganer, Jason DanielKim, SeilWagner, LudwigKim, Song-YiWalecki, PiotrKim, Su JinWalędziak, MaciejKIM, Tae-JoongWalkiewicz, KatarzynaKim, YerimWallace, Robert D.Kim, Yong HwanWallen, MatthewKim, Yong JinWalter, EdwardKim, Young RanWang, JianxiongKimer, NinaWang, LirongKing, ChristianWänman, AndersKing, Hollis H.Ward, LeighKinoshita, HiroshiWarren, JimKishida, YoshihiroWarzecha, ZygmuntKiudelis, MindaugasWaśkiewicz, ZbigniewKjellström, BarbroWaśniewska, AnnaKlar, MaximilianWatanabe, EiichiKleczynski, PawelWatanabe, YutakaKlein, BrittaWatt, PeterKleindienst, AndreaWatterson, StevenKleinstäuber, MariaWechalekar, MihirKlinger, MarcoWeems, SuzyKnoop, KathrynWeglarz, WladyslawKoch, NathalieWeiner, DanaKodama, YukiWenzel, ThomasKoestenberger, MartinWerner, TanjaKok, WouterWestmark, Cara J.Kolbenschlag, JonasWhillier, StephneyKołtuniuk, AleksandraWhite, Jeffrey D.Koltz, Michael T.Whitehead, PaulKondo, HidekazuWhitley, AdamKonger, Raymond L.Wiatrak, BenitaKönig, AlexanderWijnveld, MichielKonjar, ŠpelaWilczyński, JacekKopeć, GrzegorzWilliams, RichelleKopsaftis, ZoeWillmott, AshKorczynski, PiotrWilson, EmilijaKorolkova, Olga Y.Wingelaar, Thijs T.Korovessis, PanagiotisWinter, Max-PaulKortas, JakubWirsching, Hans-GeorgKoryak, Yuri A.Wojcik, CezaryKosmala, AleksanderWoldeamanuel, Yohannes WoubishetKothari, MohitWolf, StevenKoturbash, IgorWoo, Kyong-jeKovacs, RichardWood, Andrew JamesKowalik, ArturWoodhouse, Christopher R.J.Kowatari, RyosukeWorkman, Craig D.Kozieł, MonikaWoźniak, Magdalena MariaKozlowska, KasiaWrench, Ian JKramer, Carolyn D.Wright, Frederick A.CliveKrapohl, Björn DirkWright, Matthew DavidKraus, ArminWu, Aaron Yu-JenKrishna, RachanaWu, Chueh-HungKrogue, Justin D.Wu, Jianhua (jerry)Krol, SilkeWu, Ming-PingKrólikowska, AleksandraWysham, Katherine D.Kujan, OmarWyss, JamesKulandavelu, ShathiyahXaplanteri, PanagiotaKumagai, YouichiXiao, ZhoushengKumar, AmitXie, MinKumar, AvinashXie, RuiliKumar, DhirendraXu, JianKumar, NeerajYadav, DhananjayKumar, NitinYadav, LalitKumar, SatishYagi, ShintaroKumar, SuneelYagishita, AtsuhikoKurien, Biji TheyilamannilYamada, ShuichiKurimoto, TakujiYamagata, KenjiKuroda, NaotoYamaguchi, ShigekiKuroki, HiroshiYang, Byoung-eunKuusalo, Laura A.Yang, Jae WookKvasnica, MiroslavYang, JiulingKwiatkowska, JoannaYang, Ling LiKwon, Chan-YoungYang, YeKwon, Young SukYasaka, KoichiroLabrosciano, ClementineYazdani, AnuschLabrosse, RoxaneYehya, AminLaganà, Antonio SimoneYen, Cheng-ChiehLai, CarloYilmaz, MusaLalitkumar, Parameswaran GraceYim, JongEunLamichhane, Dirga KumarYin, KingsleyLampert, NiclasYin, WenqingLan, Hsin-ChiehYong, Su-BoonLandi, MassimoYoon, DukyongLane, Hsien-YuanYoshida, KentaroLang, MichaelYoshii, IchiroLanna, MarianoYoung, Christopher J.Lannin, Donald R.Yu, Chia-LiLanza, Gaetano AntonioYu, Yong-mingŁapińska, BarbaraZabrzynski, JanLattanzi, SimonaZacchia, MiriamLauretani, FulvioŽáček, PavelLavelli, ValentinaZakrocka, IzabelaLayden, Joseph D.Zalewski, Bartłomiej M.Lazo-Langner, AlejandroZampogna, BiagioLazzarin, GianniZanetto, AlbertoLe, Yun-zhengZange, SabineLebaron-Jacobs, LaurenceZdravković, MarkoLebwohl, Mark G.Zeballos, JoseLecomte-Grosbras, P.Zhai, KuiLedeganck, Kristien J.Zhang, KaiLee, Chien-HungZhang, YinfengLee, Chih-HungZhang, ZhenLee, Dae HoZhi, KainingLee, Ho-WonZhigalov, AlexanderLee, Jae-HyunZhou, ChuanLee, JeongshimZhurakivska, KhrystynaLee, JinwooZibis, Aristeidis H.Lee, Joo HyoungZińczuk, JustynaLee, Joon HyopZingaretti, NicolaLee, Jung-WooZiogas, Ioannis A.Lee, JunyeopZippo, Antonio GiulianoLee, Su-ShinZitta, SabineLee-Tauler, Su YeonZmarzły, NikolaLegembre, PatrickZoccali, GiovanniLéger, MaximeZorzi, AlessandroLehtihet, MikaelZotti, FrancescaLehtovirta, MikkoZuhl, MicahLehur, Paul AntoineZuk, MalgorzataLeinonen, HenriŻukow, WaleryLenders, JacquesŽulec, MirnaLensen, Sarah FrancesZullo, AngeloLenzer, BenediktZylla, Maura M.Leszczak, Justyna


